# Anti-CCP Antibody Levels Are Not Associated with MS: Results from a Case-Control Study

**DOI:** 10.1155/2015/817427

**Published:** 2015-02-02

**Authors:** Mahmut Alpayci, Aysel Milanlioglu, Veysel Delen, Mehmet Nuri Aydin, Huseyin Guducuoglu, Yasemin Bayram

**Affiliations:** ^1^Department of Physical Medicine and Rehabilitation, Faculty of Medicine, Yuzuncu Yil University, 65100 Van, Turkey; ^2^Department of Neurology, Faculty of Medicine, Yuzuncu Yil University, 65100 Van, Turkey; ^3^Department of Medical Microbiology, Faculty of Medicine, Yuzuncu Yil University, 65100 Van, Turkey

## Abstract

Citrullinated proteins have been suggested to play a critical role in the pathogenesis of multiple sclerosis (MS). Anticyclic citrullinated peptide (anti-CCP) antibody is used in the early diagnosis of rheumatoid arthritis (RA). The objective of this study was to investigate the presence of anti-CCP antibody in patients with MS compared to RA patients and healthy controls. Fifty patients with MS (38 females, 12 males; mean age 36.72 ± 8.82 years), 52 patients with RA (40 females, 12 males; mean age 40.87 ± 10.17 years), and 50 healthy controls (32 females, 18 males; mean age 38.22 ± 11.59 years) were included in this study. The levels of serum anti-CCP antibody were measured using an enzyme-linked immunosorbent assay (ELISA). The results of the study showed that anti-CCP antibody levels were significantly higher in RA patients versus MS or healthy controls (*P* < 0.001). Moreover, anti-CCP antibody was positive in 43 (83%) patients with RA, while it was negative in all MS patients as well as in all healthy controls. Also, no significant correlation was found between the anti-CCP levels and EDSS scores (*r* = −0.250). In conclusion, the results of this study did not support a positive association between serum anti-CCP antibody and MS.

## 1. Introduction

Multiple sclerosis (MS) is a chronic inflammatory autoimmune demyelinating disorder of the central nervous system (CNS) [1]. Relapsing remitting course (RRMS), that is, the most common subtype of MS, is characterized by acute attacks followed by partial or full recovery [2]. It is believed that these attacks result from acute focal inflammation of the CNS and cause disease progression, but specific factors initiating this inflammation are still unknown [1, 2]. Although the actual reason is not known precisely, genetic predisposition and environmental influences play critical roles in the development of MS, and the resulting pathology is damage of myelin [3, 4].

Myelin basic protein (MBP) is the only essential structural protein for the formation of myelin and is considered as the crucial molecule for myelination [5]. Glial fibrillary acidic protein (GFAP) is another main protein molecule that plays a key role in astrocytic functional processes [6]. It has been suggested that citrullinated CNS proteins, including GFAP and MBP, may trigger autoimmune mechanisms, contributing to MS pathogenesis [5–7]. Citrullination is a posttranslational modification resulting in the conversion of arginine to citrulline and catalyzed by peptidyl arginine deiminase (PAD) enzyme [6, 7].

While the citrullination of CNS proteins has been noted in MS, the development of antibodies to citrullinated proteins is relatively specific to rheumatoid arthritis (RA), which is a chronic inflammatory disease and often leads to peripheral joints involvement and extra-articular manifestations [8]. Anticyclic citrullinated peptide (anti-CCP), an antibody against citrullinated proteins, is frequently used as diagnostic test for RA, with a specificity of 96–100% and a sensitivity of 40–85% [9]. According to our knowledge, the anti-CCP positive rate in MS has not previously been investigated. The aim of this study was to investigate the positivity of anti-CCP in patients with MS in contrast to the patients with RA and healthy controls.

## 2. Materials and Methods

After obtaining the participants' informed consent and receiving the approval of the Ethics Committee of Yuzuncu Yil University, 50 MS patients and 52 RA patients of similar age and gender were recruited from the Department of Neurology and the Division of Rheumatology, respectively. In addition, 50 age and sex matched healthy controls were recruited from among hospital staff and relatives of patients. The characteristics of the patients and controls are presented in Table 1.

Multiple sclerosis was diagnosed according to the McDonald 2005 revised criteria [10], and RA was diagnosed according to the American College of Rheumatology (ACR) 1987 revised criteria [11]. Only patients with RRMS enrolled in the study to provide the ingroup homogeneity, and other subtypes of MS were excluded for this reason.

### 2.1. Anti-CCP Antibody Detection

The serum concentrations of anti-CCP antibodies were routinely determined by Grifols Triturus automated microenzyme-linked immunosorbent assay (ELISA) device using RA/CP-Detect Enzyme Immunoassay kit. The results was graded as negative (<12 IU/mL) or positive (≥18 IU/mL). These were consistent with the recommendation of the manufacturer (AESKULISA, Germany).

### 2.2. Statistical Analysis

Statistical analysis was performed using the Statistical Package for Social Sciences for Windows version 13.0 (SPSS Inc., Chicago, IL, USA) software program. The Kolmogorov-Smirnov test was used to confirm the normality of the distribution of continuous variables. One-way analysis of variance (ANOVA) including post hoc Duncan test and Chi-square tests had been used for comparisons. Correlation analysis was evaluated by the Pearson's correlation analysis. A *P* value of <0.05 was considered statistically significant.

## 3. Results

There were no significant differences among the three groups in terms of age (*P* = 0.121) and gender (*P* = 0.270). In RA patients, anti-CCP antibody levels were found significantly higher than in MS patients or healthy controls (*P* < 0.001). Anti-CCP antibody was positive in 43 (83%) patients with RA, while it was negative in all MS patients as well as in all healthy controls. In MS group, no significant correlation was found between the anti-CCP levels and expanded disability status scale (EDSS) scores (*r* = −0.250) (Table 1).

## 4. Discussion

There is increasing evidence that citrullination of CNS proteins, including GFAP and MBP, may play an important role in MS pathogenesis [5–7]. A recent study [6] demonstrated that high levels of citrullination are associated with areas of ongoing demyelination and revealed the presence of high levels of citrullinated GFAP in astrocytes in MS lesions. Given that astrocyte injury is thought of as an important early step in brain inflammation and GFAP is implicated in astrocytic functional processes [6], these findings support the hypothesis that citrullination is important in the pathogenesis of this demyelinating disease.

Citrulline has been found to be more frequently in the brains of patients with early-onset MS than in healthy controls, and citrullinated MBP has been reported to be increased threefold in MBP isolated from MS brain [5, 7]. Because citrullinated MBP is more susceptible to proteolytic digestion by myelin-associated proteases such as cathepsin D [6, 12], it has been hypothesized that citrullinated MBP might be contributed to the pathogenesis of MS through its increased immunogenicity [5, 7].

It is known that patients with MS have higher seroprevalence of anti-Epstein-Barr virus antibodies compared to controls [13]. It has also been reported that citrullinated Epstein-Barr nuclear antigen 1 (EBNA1) is a target of anti-CCP antibodies in RA [14]. These findings suggest a possible link between anti-CCP and MS. In addition, citrullination is considered an inflammation-dependent process [15]. Both RA and MS are Th1-related diseases, and there is an important inflammatory part in their immunopathogenetic mechanisms [16, 17]. Moreover, increased activity of PAD, which is the enzyme involved in the citrullination, has been demonstrated in synovial fluid and synovium of patients with RA and in the brain of patients with MS [7, 18]. Since the presence of anti-CCP antibodies often indicates severe joint destruction in RA [9], we hypothesized that anti-CCP antibody might also carry a prognostic or diagnostic value for MS patients.

Based on the data explained above, it can be said that there was reasonable grounds for researching a possible association between anti-CCP antibody and MS. Therefore, we wanted to investigate the presence of anti-CCP in patients with MS. Because of the high specificity (96–100%) and sensitivity (40–85%) of anti-CCP in RA [9], we included an RA cohort as well as healthy controls in the study. While the strong association of anti-CCP antibody with RA has previously been shown in many studies [8, 9], to the best of our knowledge, this is the first study investigating a possible relationship between anti-CCP and MS.

In the present study, anti-CCP antibody levels and positive rates were determined in patients with MS, RA group, and healthy controls and were compared among the three groups. As a result, the levels of anti-CCP were significantly higher in RA compared to MS or healthy controls (*P* < 0.001). The anti-CCP antibody positive rate for the RA group was 83%, whereas the rate for both MS group and healthy controls was 0%. In addition, there was no significant correlation between anti-CCP levels and EDSS scores in patients with MS (*r* = −0.250). Consequently, we failed to find evidence that anti-CCP antibody is associated with susceptibility to MS.

Most studies have demonstrated that anti-CCP antibody levels are most strongly associated with the human leukocyte antigen (HLA) region [19]. Also, there is a positive association between HLA-DR1∗15 allele and increased risk to develop MS [20]. However, the findings related to anti-CCP and this risk allele are not known. On the other hand, it has been found that HLA-DRB1∗03 and HLA-DRB1∗0901 alleles are associated with lower anti-CCP levels [19]. Since MS is associated with HLA-DR1∗15 and since MS patients are negative for anti-CCP, HLA-DR1∗15 allele may be related to anti-CCP negativity in MS.

Although anti-CCP antibodies are highly specific for RA, positive results can occur in other rheumatic and autoimmune diseases such as systemic lupus erythematosus, ankylosing spondylitis, and familial Mediterranean fever and in some infectious diseases such as brucellosis and tuberculosis [21–24]. Additionally, several citrullinated proteins (e.g., filaggrin, fibrin, vimentin, keratin, and type II collagen) can elicit anti-CCP antibodies in RA [14]. Although the mechanism of induction of anti-CCP in these diseases and the mechanism of citrullination of these proteins are not well understood, the results of our study suggest that such mechanisms may not be involved in the pathogenesis of MS.

The present study has some limitations that need to be taken into account when considering the study results. Firstly, the demonstration of the absence of anti-CCP antibodies in patients with MS may not provide sufficient information about the absence of citrullinated CNS proteins because a specific target for these antibodies is not well known [14]. Secondly, the study was performed with only patients with RRMS. For this reason, our findings may not be valid for other subtypes of MS. Thirdly, anti-CCP antibodies were measured in the serum derived from peripheral blood for this study, whereas the pathology of MS is located in CNS, and the transition of antibodies into peripheral blood might be blocked by blood-brain barrier. Nevertheless, the existence of blood-brain barrier disruption in MS pathogenesis [25] can partially reduce this limitation.

## 5. Conclusion

In conclusion, although there were some logical causes for designing this study, the study results did not indicate a positive association between serum anti-CCP antibody and MS.

## Figures and Tables

**Table 1:** Groups' characteristics and comparisons for age, gender, and anti-CCP.

	MS group (*n* = 50)	RA group (*n* = 52)	Control group (*n* = 50)	*P* value
Age (years, mean ± SD)	36.72 ± 8.82	40.87** ± **10.17	38.22** ± **11.59	0.121
Gender (female, %)	38 (76%)	40 (77%)	32 (64%)	0.270
Anti-CCP levels	3.29 ± 1.92^a^	106.84 ± 106.82^b^	3.20 ± 1.67^a^	0.001
Anti-CCP (positive, %)	0 (0%)	43 (83%)	0 (0%)	
EDSS scores	2.37 ± 1.57			
Correlation (anti-CCP and EDSS)	−0.025			

MS, multiple sclerosis; RA, rheumatoid arthritis; anti-CCP, anticyclic citrullinated peptide.

Note: when compared with the RA group, ^a^ *P* < 0.001; when compared with the control group or the MS group, ^b^ *P* < 0.001.
